# International rates of receipt of psychological therapy for psychosis and schizophrenia: systematic review and meta-analysis

**DOI:** 10.1186/s13033-023-00576-9

**Published:** 2023-03-31

**Authors:** Susanna Burgess-Barr, Emily Nicholas, Bethany Venus, Niharika Singh, Abigail Nethercott, Gemma Taylor, Pamela Jacobsen

**Affiliations:** 1 Department of Psychology, University of Bath, Bath, BA2 7AY UK; 2 Department of Psychology, University of Derby, Kedleston Road, Derby, DE22 1GB UK

**Keywords:** Psychotic disorders, Cognitive behaviour therapy, Meta-analysis, Psychosocial interventions, Evidence-based medicine

## Abstract

**Background:**

International clinical practice guidelines commonly recommend the provision of psychological therapies for psychosis and schizophrenia as an adjunct to medication. However, access to recommended therapies in routine clinical practice is limited. The aim of this review was to synthesise the available data on the provision of recommended psychological therapies for psychosis and schizophrenia across international mental health systems.

**Methods:**

Electronic databases (PsychINFO, Pubmed and EMBASE) were searched for audits, service evaluation projects, or surveys, which reported data on rates of offer or receipt of any recommended psychological therapy or therapeutic intervention as part of routine clinical care.

**Results:**

Twenty-two eligible studies from 9 countries were identified (*N* participants = 79,407). The most commonly recommended therapies in national guidelines were Cognitive-Behavioural Therapy for Psychosis (CBTp) and Family Interventions (FI). The overall pooled prevalence of rate of receipt of CBTp was 24% [95% CI 0.15–0.32] based on 15 studies (*N* = 42,494), with a higher rate of receipt of therapy found when pooling data from Early Intervention services only (41% [95% CI 0.21–0.60], 6 studies, *N* = 11,068). The overall pooled prevalence of rate of receipt of FI was 30% [95% CI 0.22–0.37] based on 14 studies (*N* = 13,863).

**Conclusions:**

Overall rates of receipt of recommended psychological therapies for psychosis were low across the 9 countries data were available for in this review. However, there were high rates of heterogeneity across studies, meaning that pooled estimates should be interpreted with caution. Sources of heterogeneity included different service settings (e.g. early intervention vs. non-early intervention services), and varying methods used to collect the data (e.g. audit of electronic health records vs. self-report etc.). There were no available data from the continents of South America, Asia, or Africa, meaning that a truly global picture of provision of psychological therapies for psychosis and schizophrenia is currently lacking.

**Supplementary Information:**

The online version contains supplementary material available at 10.1186/s13033-023-00576-9.

## Introduction

Schizophrenia-spectrum disorders are characterised by core symptoms of psychosis including delusions, hallucinations, and thought disorder [1]. Schizophrenia is one of the top 10 leading causes of disability worldwide [2]. It is characterised by high rates of relapse and symptoms which can often persist across the lifespan [3]. People with schizophrenia and psychosis may receive care in a variety of settings including acute psychiatric wards, rehabilitation settings, and community mental health teams. Due to the complexity of service users’ needs, care is usually provided by a multi-disciplinary team including psychiatrists, mental health nurses, occupational therapists, social workers, and psychologists.

Clinical guidelines are significant drivers of national health policies and commissioning of services across international mental health systems. A 2011 review of international schizophrenia guidelines [4] focused on 5 guidelines including those from Australia/New Zealand (RANZCP; Royal Australian and New Zealand College of Psychiatry), United States of America (APA; American Psychiatric Association & PORT; Patient Research Outcomes Team), Germany (DGPPN; German Society of Psychiatry, Psychotherapy and Nervous Diseases) and the United Kingdom (NICE; National Institute for Clinical and Health Excellence)). All of these guidelines recommended psychosocial interventions as an adjunct to medication. There were some minor differences between guidelines in terms of recommended psychological therapies, but the therapies which were universally recommended were Cognitive-Behavioural Therapy for Psychosis (CBTp) and/or Family Interventions (FI).

The inclusion of psychological therapies as routinely recommended treatments for schizophrenia reflects a significant policy shift away from a solely pharmacological treatment approach, given the evidence base for psychological therapies in reducing distress and impairment associated with symptoms, and promoting recovery e.g. [5, 6]. In contrast to medical interventions, data regarding the implementation of psychological interventions is limited. For example, in a Cochrane review examining the efficacy of guideline implementation strategies only 2 out of 6 studies included data relating to psychological interventions [7]. A major charity commission in the United Kingdom (UK) found that service users often experience high levels of dissatisfaction with their care within schizophrenia/psychosis pathways and frequently reported inadequate support for families and carers, and lack of access to recommended psychological therapies [8]. Further evidence for limited access to therapies comes from a systematic review which reported implementation rates of between 4 and 100% for CBTp and 0–53% for FI, based on 11 UK-based studies [9]. The large variation in reported implementation rates arose due to sampling differences, and different methods used for assessing implementation rates across studies. For example, some studies used approaches which would be more affected by response bias, such as self-selecting service users responding to a charity survey. Other studies relied on staff report of receipt of therapies, rather than more robust methods such as independent auditing of electronic health records.

Evidence from other countries indicates that inadequate implementation of clinical guidelines for the provision of psychological therapies is not a UK problem only. For example, a recent review of schizophrenia guidelines across 12 countries in South-East Europe (including Croatia, Greece, and Serbia) found that although most recommended psychological therapies including CBTp and FI, they were poorly implemented in routine care, often due to a lack of trained staff [10]. Comprehensive data on actual rates of receipt of therapy is not available for every country which has schizophrenia treatment guidelines. Some studies have used proxy measures to assess clinical guideline implementation such as availability of trained clinicians. A study using this approach estimated the accessibility of CBTp in the USA and Canada by using a national survey of workforce training and reference to known prevalence rates of schizophrenia [11]. The findings suggested that only 0.57% of the mental health workforce were CBTp trained, representing between 11.5 and 22.8 CBTp trained clinicians per 10,000 people with a schizophrenia/psychosis disorder. Based on this, the authors concluded that recommended psychological therapies remain largely inaccessible to service users in North America.

In summary, psychological therapies for psychosis are now routinely recommended in international clinical guidelines, but service users may not be able to access these therapies due to low rates of implementation in routine clinical practice. The only previous systematic review on implementation of clinical guidelines for psychological therapies for schizophrenia/psychosis was based on UK studies only [9], meaning that a global picture of implementation is lacking. The current review aimed to fill that gap by searching for and synthesising available international data on implementation of evidence-based psychological therapies for schizophrenia/psychosis. This addresses a question of high importance to service users and carers, alongside mental health clinicians and healthcare commissioners, in terms of ensuring fair access to evidence-based therapies.

## Method

### Review question

What are international rates of receipt of nationally recommended psychological therapy for psychosis?

### Registration of review protocol

We wrote a review protocol and registered it on the Open Science Framework (https://doi.org/10.17605/OSF.IO/FSEQM; date uploaded 2nd December 2020) and the online Prospero database (https://www.crd.york.ac.uk/PROSPERO; CRD42020224002; date registered 14th December 2020). This review is reported in line with Preferred Reporting Items for Systematic Reviews and Meta-analyses (PRISMA) guidelines [12].

### Searches

We searched for relevant peer reviewed journal articles in electronic databases (PsychINFO, Pubmed and EMBASE) published from 1st January 2010 up until 27th November 2020 (the date the initial searches were run). The searches were then updated on 21st November 2022. The rationale for this time frame was to give a comprehensive picture as possible of current practice, whilst also allowing for the effect of updating of recommendations in line with new research evidence accumulating over time. See Additional file 1 for a complete list of search terms. We were already aware of two government reports related to UK data therefore a basic internet search using comparable search terms was conducted in an attempt to identify corresponding reports for different countries (‘Identification of new studies via other methods’ in PRISMA diagram).

### Inclusion/exclusion criteria

#### Study design

Audits, service evaluation projects, surveys.

#### Setting

Any adult (18+) mental health team or service, or early intervention service open to both under and over 18s.

#### Language

Any (Google translate was used where necessary to assess eligibility for papers published in languages other than English).

#### Participants

Adults (> 18 years) with any psychosis spectrum disorder as defined by the 10th revision of the International Statistical Classification of Diseases and Related Health Problems (ICD-10) codes (F20-29) [13] or any schizophrenia spectrum disorder as defined by the Diagnostic and Statistical Manual of Mental Health Disorders 5th edition (DSM-5), or previous versions of these diagnostic manuals where relevant [14]. Studies involving participants with a mixed age range including some < 18 years were also included.

#### Intervention

Studies reporting observed rates of offer, referral, or receipt of any recommended psychological therapy or therapeutic intervention delivered as part of routine clinical care or service evaluation project (i.e. not as part of a clinical trial, or other study involving randomisation to condition). We referred to relevant national guidelines to help determine whether the inclusion criteria of being ‘recommended’ (in the country where the study was conducted) was met (e.g. National Institute for Health and Care Excellence (NICE) guidelines for UK based studies). Studies relating to countries where national guidelines on recommended therapies were not available were included where they reported on interventions which were present in other guidelines.

#### Outcomes

Papers reporting proportions of service users being offered and/or receiving recommended psychological interventions.

### Study selection and data extraction

**Fig. 1:**
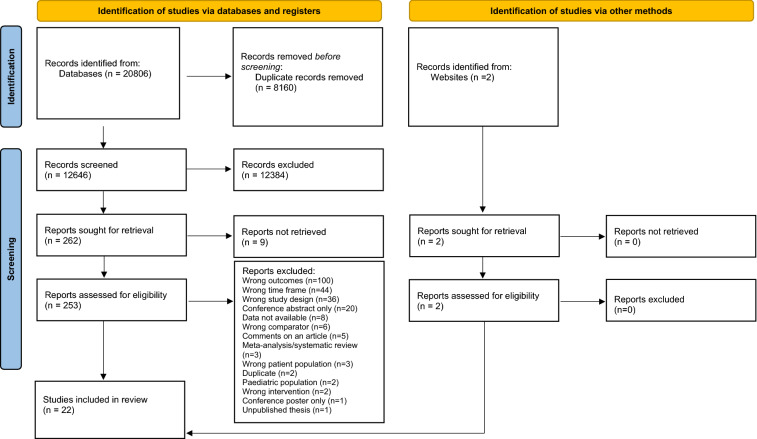
PRISMA Diagram

All studies were independently double screened by two reviewers at both title/abstract and full-text stage using the systematic review software Covidence (https://www.covidence.org/). Any discrepancies were resolved by discussion to reach consensus, with consultation with the senior author where needed to reach a final decision. We contacted corresponding authors to ask for additional information needed to assess eligibility where necessary. For summary of searches see PRISMA diagram (Fig. 1). We extracted data on the number of service users being offered and/or receiving recommended psychological interventions (numerator), and the size of the total sample (denominator) in order to calculate a pooled estimate of proportions across studies. We also extracted data where available on potential predictor variables of therapy receipt including age, ethnicity, diagnosis, gender, marital status, and service type. All data was independently double extracted by two reviewers using a standardised template.

### Quality assessment

A modified version of the Joanna Briggs Institute Critical Appraisal Checklists for Studies Reporting Prevalence data was used to assess the quality of studies [15] as recommended for this type of review [16]. For the purposes of this review, we removed Question 3 (Was the sample size adequate?) as this was difficult to apply to routine clinical settings where the sample size was predetermined due to the size of the service being audited. The tool was further modified to include a scoring system to facilitate the comparison of studies. Studies were scored as follows on the 8 remaining questions: Yes = 2, No = 1, Unclear = 0. Final scores were then presented as percentages (with the denominator adjusted where relevant if any questions were assessed as not relevant to a particular study). Quality assessment was single-rated, with a random sample (6/22; 27%) double-checked by the senior author for accuracy.

### Data analysis

The proportion of service users being offered or receiving a recommended therapy was calculated using the following formula:-

$$\left(\frac{\mathrm{People \,who \,received \,recommended \,psychological \,therapy }}{\mathrm{People \,in \,the \,service}/\mathrm{sample}}\right)*100$$

Analyses were performed separately for offer and receipt of therapy. A pooled estimate of proportions was calculated using a random effects model using the ‘Metaprop’ package in Stata [17]. This model ensures that the combined estimate captures the range of populations present across studies, rather than weighting studies solely by sample size, as individual studies regardless of size may contain information regarding a population that no other study has captured [18]. Pooled estimates were depicted graphically using a forest plot. Heterogeneity was examined using the I^2^ statistic. We aimed to run additional analyses on possible predictors of therapy receipt based on demographic or clinical characteristics (age, ethnicity, diagnosis, gender, marital status, and service type) where data were available. Sensitivity analyses were conducted by pooling prevalence rates from EI studies alone for both receipt of CBTp and FI as well as for studies referring to data from countries with defined treatment guidelines compared with countries where no treatment guidelines were available.

## Results

### Study selection

A total of 20,806 records were identified from database searches, and 12,646 were screened at title/abstract stage after duplicates were removed; 262 reports were identified as potentially eligible and sought for retrieval; 253 records were screened at full-text review plus an additional 2 records which were identified via other sources; a further 233 studies were excluded after this stage, with a total of 22 unique studies being identified as eligible for inclusion in the review. See Fig. 1 (PRISMA diagram) for a summary of how studies were selected.

### Overview of study design and characteristics

See Table 1 for characteristics of the 22 included studies. Included studies came from 9 different countries, all from the continents of Europe, North America, or Australia (United Kingdom (n = 9), United States of America (n = 4), Canada (n = 3), Australia (n = 1), Ireland (n = 1), Portugal (n = 1), France (n = 1), The Netherlands (n = 1), Spain (n = 1)). Ten of the 22 studies were from Early Intervention teams with the remainder including a range of clinical settings including recovery teams, community mental health teams, public services, individuals following discharge from hospital, inpatient units, and outpatient clinics. Studies reported data mainly collected through audits of healthcare records or service user/staff surveys; however, many studies did not provide specific details of how data were collected. Sample sizes ranged between 35 and 35,812 people. Studies reported data for service users receiving Cognitive Behavioural Therapy for Psychosis (CBTp) (n = 16), Family Interventions (FI) (n = 14), Psychotherapy (n = 1) and Cognitive Remediation Therapy (n = 2). Most studies reported data from over a 12-month period (n = 15). Other time frames included 6 months (n = 1), 2 years (n = 1), 3 years (n = 4), 4 years (n = 1), 10 years (n = 1) and not specified (n = 1).

**Table 1:** Characteristics of included studies

Study	Country & Clinical Guideline	Setting	Methods	N	Audit period	Diagnosis	Treatment description
Addington et al. [35]	Canada Canadian Psychiatric Association	2 hospital outpatient clinics and 1 community mental heath clinic	Medical record review	216	2010–2011	Schizophrenia, schizoaffective disorder + comorbid substance use disorder	Individual and/or group therapy + Family treatment, FI minimum 4 sessions
Bedard et al. [49]	Canada Canadian Psychiatric Association	Early Intervention	Audit of care pathway forms	108	’12 month time period’	First episode psychosis	FI, psychoeducation
Bioque et al. [33]	Spain Catalan Agency for Health Technology, Assessment and Research	Multicenter, mostly tertiary University Hospitals	Treatment was recorded at each assessment visit	119	10.2012–12.2015	Schizophrenia or schizophreniform disorder	CBT + FI + Cognitive Remediation
Breitborde et al. [19]	United States of America APA; American Psychiatric Association & PORT; Patient Research Outcomes Team	Early Intervention	Audit, not specified	68	6 months	Schizophrenia spectrum disorder or affective disorder with psychotic features	CBTp + FI + Metacognitive remediation therapy
Clarke et al. [20]	Ireland HSE; Health Service Executive	Community-based mental health service, patients presenting to one of four General Adult Sectors with first episode psychosis	Audit, not specified	66	2002–2012	Schizophrenia, acute and transient psychotic episode, psychosis, drug-induced psychosis, mania with psychotic symptoms, severe depression with psychosis, delusional disorder, schizoaffective disorder	CBT + FI, family behavioural therapy and family education
Coentre et al. [30]	Portugal No national schizophrenia clinical guideline	Early Intervention	Audit, not specified	39	09.2017–09.2018	Schizophrenia, brief psychotic disorder, psychotic disorder not otherwise specified, major depressive disorder with psychotic features, bipolar disorder type 1 manic episode, cannabis induced psychotic disorder	CBTp
Coleman et al. [36]	United States of America APA; American Psychiatric Association & PORT; Patient Research Outcomes Team	Healthcare systems participating in the Mental Health Research Network	Audit of insurance claims and electronic medical record databases	35,812	2010–2011	Schizophrenia spectrum disorder + other psychosis	Psychotherapy
Colling et al. [29]	United Kingdom (England only) NICE; National Institute for Clinical and Health Excellence	Early Intervention + Promoting Recovery	Audit of electronic health care records	2579	07.2012–07.2013	Schizophrenia, schizoaffective disorder + other schizophrenia spectrum disorder	CBTp, at least one session
Cotter et al. [50]	United Kingdom (England only) NICE; National Institute for Clinical and Health Excellence	Early Intervention	Audit, not specified	165	04.2012–03.2013	First episode psychosis	FI, uptake in first 3 months in service
Dubreucq et al. [32]	France No national schizophrenia clinical guideline	Stabilised outpatients recruited from FondaMental Advanced Centers of Expertise for Schizophrenia cohort	Audit, not specified	183	Baseline + 1 Year follow up	Schizophrenia + schizoaffective disorder	CBTp + Cognitive remediation therapy
Fischler et al. [24]	Canada Canadian Psychiatric Association	326-bed public teaching hospital specializing severe mental illness	Audit, not specified	326	04.2014–03.2015	Schizophrenia + schizoaffective disorder	CBTp
Greenfield et al. [27]	United Kingdom (England only) NICE; National Institute for Clinical and Health Excellence	Early Intervention (over 35’s only)	Review of electronic health records + discussion with clinicians	72	2011–2014	Schizophrenia, schizoaffective disorder, manic psychosis, depressive psychosis, PTSD, organic psychosis, drug induced psychotic disorder	CBTp, ‘formal in past year’
Haddock et al. [23]	United Kingdom (England only) NICE; National Institute for Clinical and Health Excellence	Community Mental Health teams	Audit of electronic records	187	11.2009–11.2010	Schizophrenia spectrum disorder	CBTp + FI
Harvey et al. [28]	Australia RANZCP; Royal Australian and New Zealand College of Psychiatry	Public specialised mental health services, non-government organisations, clinical services	Service user survey	1825	03.2009–03.2010	Psychosis	CBTp, ‘evidence-based level’ at least 8 sessions + FI, family psychoeducation at least 6 sessions
Johns et al. [22]	United Kingdom (England only) NICE; National Institute for Clinical and Health Excellence	Promoting Recovery + Early Intervention	Audit of self-report + electronic health records	6369	11.2012–10.2015	Schizophrenia spectrum, bipolar, psychotic depression and other (psychosis)	CBTp, started by end of referral period
Mason et al. [34]	United Kingdom (England only) NICE; National Institute for Clinical and Health Excellence	Large secondary care mental healthcare provider	Audit of electronic healthcare records	20,078	01.2007 – 06.2020	ICD-10-defined schizophrenia spectrum disorder (F20–F29)	CBTp
Molag et al. [31]	The Netherlands Trimbos Institute	Flexible Assertive Community Treatment (FACT) teams	Audit, not specified	60	2012 – not specified	Schizophrenia	CBTp + FI
North et al. [51]	United States of America APA; American Psychiatric Association & PORT; Patient Research Outcomes Team	Early Intervention	Audit of monthly service use data collected from billing records	35	02.2015 – 03. 2016	Schizophrenia, schizoaffective disorder, bipolar disorder (with psychosis), major depressive disorder (with psychosis)	FI, at least monthly family peer recovery support services
Oluwoye et al. [37]	United States of America APA; American Psychiatric Association & PORT; Patient Research Outcomes Team	Early Intervention	Audit, not specified	211	2015–2019	Schizophrenia-spectrum disorder + other psychotic disorders including delusion disorder	FI, at least one session during 24-month period
Rathod et al. [21]	United Kingdom (England only) NICE; National Institute for Clinical and Health Excellence	Early Intervention	Audit of routinely collected clinical data	124	2017–2018	Not stated	CBTp + FI, taken up within 6 months
Royal college of psychiatrists, [25]	United Kingdom (England only) NICE; National Institute for Clinical and Health Excellence	Early Intervention	Service user survey to random sample	10,560	2019–2020	First episode psychosis	CBTp + FI, at least one session
Royal college of psychiatrists, [26]	United Kingdom (Wales only) NICE; National Institute for Clinical and Health Excellence	Early Intervention	Service user survey to random sample	205	2019–2020	First episode psychosis	CBTp + FI, at least one session

### Quality assessment

The overall quality of included studies was good, with the majority of studies (17/22) scoring above 75% (Table 2). The lowest scoring studies were Breitborde et al. [19] (63%) and Clarke et al. [20] (50%) largely due to issues regarding unclear methods of data collection and sampling.

**Table 2:** Quality assessment data

Study	Was the sample frame appropriate to address the target population?	Were study participants sampled in an appropriate way?	Were the study subjects and the setting described in detail?	Was the data analysis conducted with sufficient coverage of the identified sample?	Were valid methods used for the identification of the condition?	Was the condition measured in a standard, reliable way for all participants?	Was there appropriate statistical analysis?	Was the response rate adequate, and if not, was the low response rate managed appropriately?	Raw score	Score (%)
Addington et al. [35]	Y	Y	Y	Unclear	Y	Y	Y	N	13/16	81
Bedard et al. [49]	Y	Y	N	Y	Y	Unclear	Y	N/A	11/14	79
Bioque et al. [33]	Y	Y	Y	Unclear	Y	Y	Y	Y	14/16	88
Breitborde et al. [19]	Y	N	Y	Unclear	Y	Unclear	Y	N	10/16	63
Clarke et al. [20]	Y	N	Y	Unclear	Unclear	Unclear	Y	N/A	7/14	50
Coentre et al. [30]	Y	Y	Y	Y	Unclear	Unclear	Y	Y	12/16	75
Coleman et al. [36]	Y	Y	N	Y	Y	Unclear	Y	N/A	11/14	79
Colling et al. [29]	Y	Y	Y	Y	Y	Y	Y	N/A	14/14	100
Cotter et al. [50]	Y	Y	Y	Y	Unclear	Y	Y	N/A	12/14	86
Dubreucq et al. [32]	Y	Y	Y	Y	Y	Y	Y	Y	16/16	100
Fischler et al. [24]	Y	Y	N	Y	Y	Unclear	Y	N/A	11/14	79
Greenfield et al. [27]	Y	Y	Y	Y	Unclear	Unclear	Y	N/A	10/14	71
Haddock et al. [23]	Y	Y	Y	Unclear	Y	Y	Y	N/A	12/14	86
Harvey et al. [28]	Y	Y	Y	Y	Y	Unclear	Y	Y	14/16	88
Johns et al. [22]	Y	Y	Y	Y	Y	Unclear	Y	N/A	12/14	86
Mason et al. [34]	Y	Y	Y	Y	Y	Y	Y	N/A	14/14	100
Molag et al. [31]	Y	Y	Y	Y	Unclear	Unclear	Y	N/A	10/14	71
North et al. [51]	Y	Y	Y	Y	Y	Unclear	Y	N	13/16	81
Oluwoye et al. [37]	Y	Y	Y	Y	Y	Y	Y	N/A	14/14	100
Rathod et al. [21]	Y	Y	Y	Y	Unclear	Unclear	Y	N/A	10/14	71
Royal college of psychiatrists [25]	Y	Y	Y	Y	Y	N	Y	Y	15/16	94
Royal college of psychiatrists [26]	Y	Y	Y	Y	Y	N	Y	Y	15/16	94

### Quantitative synthesis of prevalence of offer/receipt of therapy: meta-analysis

#### Cognitive-behavioural therapy for psychosis (CBTp)

Three studies reported data for both proportions of service users being (i) *offered* and (ii) *receiving* (CBTp) within the same sample [21–23]. The distinction between offer and receipt is important, as not everyone who is offered therapy may be expected to take up the offer. Two studies reported low rates of both offer and receipt of CBTp in Community Mental Health Teams in the UK, with only a small gap between the prevalence rates for offer and receipt (11% vs. 6.3% respectively [22]; 6.9% vs. 5.3% [23]). In contrast, a study reporting data solely from Early Intervention teams (which provide care for people for a time-limited period of time after a first episode of psychosis) reported a similar rate of receipt of CBTp (6.5%) but a much higher rate of offer of CBTp (67.7%) [21]. One additional study reported data solely on offer of CBTp [24] but not receipt, whilst 12 studies reported data solely on receipt of CBTp, but not offer [19, 20, 25–34]. This perhaps reflects the added difficulties in assessing whether someone has been offered therapy, as this may not be formally recorded in the same way as attendance at therapy sessions etc. which can be more easily audited through clinical notes.

**Fig. 2:**
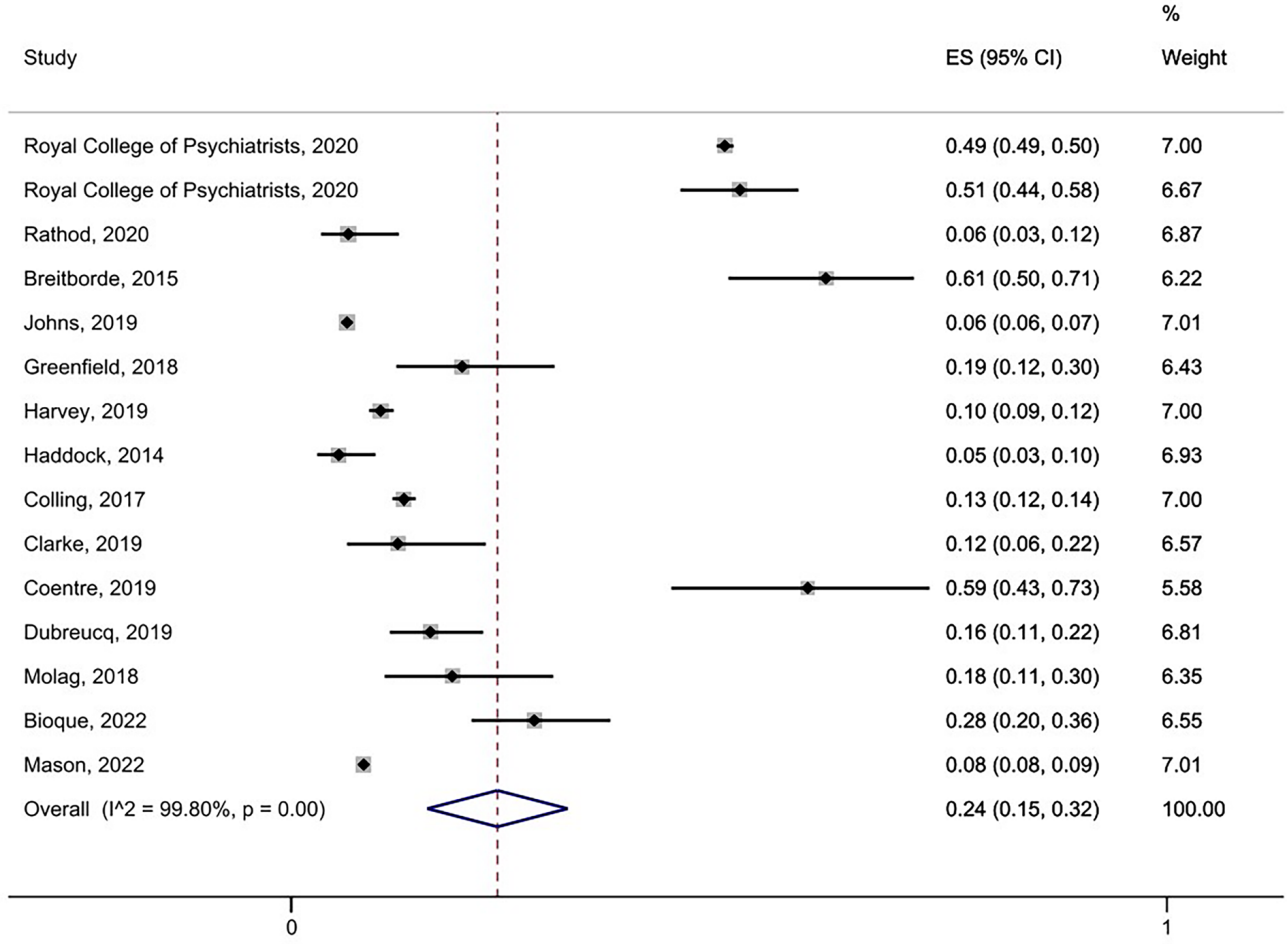
Pooled prevalence of service-users who received Cognitive Behavioural Therapy for psychosis (CBTp)

For all the studies which reported data on service users being *offered* CBTp (*k* = 4, n = 7006) a random effects model yielded a pooled prevalence rate of 23% (95% CI 0.11–0.35). See Additional file 2: Fig. S1) for forest plot. The pooled prevalence rate for service users *receiving* CBTp (*k* = 15, n = 42,494) was 24% (95% CI 0.15–0.32; see Fig. 2). Heterogeneity was high in both models (I^2 = 98.4% & 99.8% respectively). We ran a sensitivity analysis to compare the prevalence rate for studies where guidelines were clearly defined (*k* = 13, n = 42,272) with the rate for the two studies where no treatment guidelines were found [30, 32], (*k* = 2, n = 222). The random effects model showed a prevalence rate of 22% (95% CI 0.13–0.30) and 20% (95% CI 0.15–0.25) respectively, which were both comparable to the pooled prevalence rate for all 15 studies together (24%). See Additional file 2: Figs. S2 and S3 for forest plots).

We observed that studies reporting data from Early Intervention (EI) settings appeared to show higher rates of therapy receipt compared to non-EI settings. We ran a sensitivity analysis by pooling prevalence rates from EI studies alone reporting data on receipt of CBTp (*k* = 6, n = 11, 068). The random effects model showed a pooled prevalence rate of 41% (95% CI 0.21–0.60), which was higher than the pooled prevalence rates for all studies combined (24%) indicating that on average rates of receipt are higher in EI compared to non-EI settings (see Additional file 2: Fig. S4 for forest plot).

### Family intervention (FI)

**Fig. 3:**
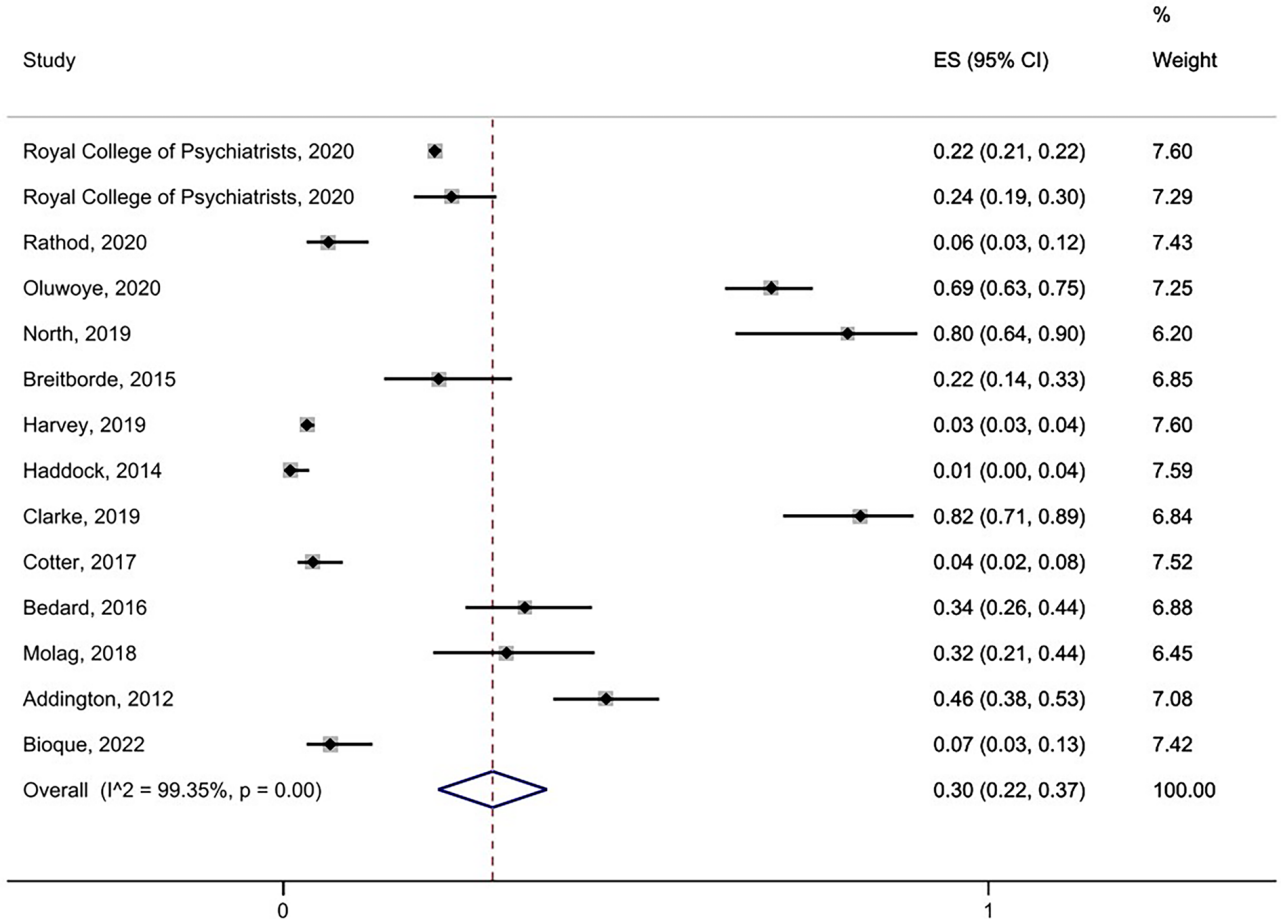
Pooled prevalence of service-users who received Family Intervention (FI)

Only two studies reported prevalence rates of service users being *offered* FI. These were Rathod et al. [21] who reported a rate of 64.5% (80/124 service users) and Haddock et al. [23] who reported a rate of 1.6% (3/187 service users). As noted earlier, this large difference in rates is likely to reflect differences in the clinical setting, with Rathod et al. reporting data from an Early Intervention service and Haddock et al. reporting data from general Community Mental Health Teams. Fourteen studies reported prevalence rates of service users *receiving* FI (n = 13,863). The random effects model showed a pooled prevalence rate of 30% (95% CI 0.22–0.37; see Fig. 3). Heterogeneity was very high (I^2 = 99.4%).

We similarly ran a sensitivity analysis on studies reporting data on receipt of Family Interventions (FI) from Early intervention (EI) settings only (*k* = 8, n = 11,476). The random effects model yielded a pooled prevalence rate of 32% (95% CI 0.20–0.44), which was the same as for the pooled prevalence rate for all 14 studies together including both EI and non-EI services (30%). See Additional file 2: Fig. S5) for forest plot.

### Other recommended therapies

Five studies [19, 33, 35, 36] reported rates of receipt of other recommended therapies including metacognitive remediation therapy, psychotherapy, and cognitive remediation therapy (see Additional file 2: Table S1 for summary).

### Narrative synthesis of predictors of offer/receipt of therapy

Data was extracted where available for predictors of therapy receipt. Due to significant differences across studies in how data were collected and reported (for example, different categorisation of age brackets) it was not possible to statistically pool results across studies in a meaningful way. We focused therefore on a narrative synthesis of these findings (see Additional file 2: Table S2 for summary).

Age was investigated as a possible predictor of the receipt of therapy by five studies, with four of these studies reporting data relating to receipt of CBTp [23, 28, 29, 32, 34] and one relating to Family Interventions [37]. Colling et al. [29] reported a statistically significant effect of age on receipt of therapy (CBTp), reporting that in their sample, under 41s were more likely to have received CBTp than over 41s (odds ratio (OR) = 1.57; 95% CI 1.01–1.72). Mason et al. [34] reported a Welch two sample t-test which found significant between-group differences in age (t = 15.34, p < 0.01), where those who had received CBTp had a lower mean age (M = 33.12 SD = 11.5) compared with those who did not (M = 35.88, SD = 13.08). Harvey et al. [28] reported that females in their study (conducted in Australia) were more than twice as likely to receive CBTp than males (OR = 2.21; 95% CI 1.60–3.05). However, three UK studies reported no statistically significant effect of gender on likelihood of therapy receipt [23, 29, 34]. Two of these UK studies also reported data on ethnicity as a possible predictor of receipt of CBTp. Haddock et al. reported no statistically significant effect, whereas Colling et al. reported that White service users were more likely to receive CBTp compared to Black service users (OR = 1.43; 95% CI 1.10–1.85). A USA study reported no statistically significant effect of ethnicity on likelihood of receipt of Family Interventions in an Early Intervention setting [37].

Four studies reported diagnosis as a significant predictor of therapy receipt. Harvey et al. [28] reported that service users with non-affective psychosis were more likely to receive CBTp compared to service users with affective psychosis (OR = 2.51; 95% CI 1.79–3.52). Haddock et al. [23] reported that service users with a diagnosis of ‘other psychosis’ were more likely to receive CBTp compared to schizophrenia (OR = 3.75), with Colling et al. [29] reporting similar findings. Mason et al. [34] reported significant effects of having a comorbid diagnosis of depression (χ2 = 87.36), bipolar (χ2 = 71.94) or anxiety (χ2 = 118.28). Colling et al. [29] also reported service type as being a significant predictor of therapy receipt with service users in Early Intervention (EI) teams more likely to receive CBTp than service users in non-EI teams (OR = 1.98; 95% CI 1.40–2.81).

## Discussion

This systematic review and meta-analysis aimed to synthesise the available data on international rates of receipt of recommended psychological therapies for psychosis in routine clinical practice. We also aimed to summarise the available evidence on predictors of receipt of psychological therapy such as service type, age, and ethnicity. We found data from 9 different countries within 3 continents (Europe, North America, Australia), with many eligible studies being from the UK (9/22). All were high income countries according to World Bank classifications. Overall, this indicates a lack of available data from low- and middle-income countries, and from high-income countries outside of the UK, and especially from the continents of South America, Africa, and Asia which were not represented in our sample at all.

Overall, our results indicate low rates of both offer and receipt of recommended therapies (CBTp offered = 23% [95% CI 0.11–0.35], CBTp received = 24% [95% CI 0.15–0.32], FI received = 30% [95% CI 0.22–0.37]). When data was pooled from Early Intervention (EI) services only, rates of CBTp receipt was found to be higher (41% [95% CI 0.21–0.60]) but similar for FI (32% [95% CI 0.20–0.44]). It is important however to note the high level of heterogeneity between studies, which means that pooled estimates should be interpreted with caution. Only 5/22 studies reported any data on clinical and demographic characteristics which might be predictors of likelihood of therapy receipt. These data could not be meaningfully combined in a quantitative synthesis due to differences in how factors were defined and how data were reported between studies. A narrative synthesis indicated no consistent findings on the effect of age, gender, ethnicity, diagnosis, or marital status on therapy receipt. Colling et al. [29] and Mason et al. [34] both reported significant effects of age indicating that younger people were more likely to receive therapy than older people; this is likely due to the fact that receipt of therapy was more common in EI services than non-EI, and EI service users are usually younger due to the onset of a first episode commonly occurring in late adolescence/early adulthood.

Despite psychological therapies being recommended as evidence-based interventions alongside medication in international clinical guidelines, their availability lags far behind medication according to the findings of this review. Medication is almost always available to people with a diagnosis of schizophrenia, although discontinuation rates are high [38]. In contrast the current data indicates only around a third of service users receive internationally recommended therapies (CBTp and FI), with high levels of variation both within and between countries. The absence of clearly defined treatment guidelines did not appear to influence prevalence rates, however due to the small number of studies where this was the case (n = 2), it is difficult to draw any substantial conclusions. The results of this review are broadly consistent with the findings of the previous review by Ince et al. [9] which was focused on UK-based studies only, and reported rates of receipt of CBTp from 4 to 100%. The wide range of different rates of therapy receipt in both the current and the Ince review likely arose due to similar factors such as differences between studies in the criteria used to determine offer or receipt of therapy and differing methods of data collection.

Our findings indicated higher levels of implementation of CBTp in Early Intervention (EI) services compared to all service types pooled together. This may reflect policies in some countries which aim to optimise the care people receive when they experience a first episode of psychosis, to maximise the chance of a good recovery and to preserve personal, social, and occupational functioning as much as possible. For example, in the UK National Health System (NHS) a new access and waiting time standard for early intervention in psychosis services was introduced in 2016, meaning that at least 50% of people experiencing a first episode of psychosis must start treatment within 2 weeks of referral, and treatment must be in line with NICE (National Institute for Clinical and Health Excellence) guidelines. The Early Intervention model for first episode psychosis is becoming more widespread internationally, which may lead to increased access to psychological therapies for people in these services. For example the NAVIGATE program which was initially developed in the USA for people with first episode psychosis is now being rolled out in Israel [39]. However, despite the rapid proliferation of coordinated speciality care to improve outcomes for people experiencing a first episode of psychosis, access to psychological therapies for people outside of early intervention services may lag behind based on the findings of this review.

In order to improve access to recommended psychological therapies, it is important to understand barriers and facilitators to implementation. Previous reviews which have synthesised the available data on barriers to guideline implementation for CBTp and FI, have shown that barriers arose at multiple levels including organisational, staff, and service user levels [9, 40, 41]. These included negative staff attitudes towards referring service users for therapy, lack of specialised training available for staff to deliver the therapy, and dominance of a biological model of care [42]. Similar findings were reported from a study of staff attitudes, social norms, and behavioural control in Canada and Australia, with survey data suggesting that these staff factors significantly predicted CBTp delivery in practice [43].

In terms of strengths and limitations of this study, we followed best practice in the conduct of systematic reviews and meta-analyses according to Cochrane review standards. This included writing and pre-registering a comprehensive review protocol, keeping an audit trail of any subsequent protocol changes, and double-rating all records at both title/abstract and full-text stages. Our searches returned over 10,000 records indicating a comprehensive search; however relevant studies could have been missed given the complexities of writing effective search teams for such a broad topic. We did not search grey literature on the basis that the data we were looking for would most likely be found in the peer-reviewed literature, however this again may have led to relevant papers being missed. Our inclusion criteria specified studies which reported data on interventions included in treatment guidelines, however, it is possible that relevant data may have been missed where studies reported on therapeutic interventions that were not clearly defined and therefore not identifiable as ‘recommended’ e.g. [44, 45]. Prior to the study we were aware of government reports that gave relevant data in the UK, however, we were unable to find equivalent data in other countries which also could have been missed.

Although overall the methodological quality of the included studies was high, there was a wide range of methods and clinical settings included across studies, making a coherent synthesis more challenging. For example, some studies used more robust methods of assessing offer and receipt of therapy such as independent reviewing of electronic health records using key search terms e.g. Colling et al. [29]. Other studies used methods more open to response bias such as inviting service users with psychosis for interviews where not all eligible people took part [28]. There was also a considerable amount of variation across studies in terms of how interventions were defined with regards to therapy content, number of sessions, clinicians delivering intervention etc. The NICE guidelines in the UK for example recommend that CBTp be delivered over at least 16 sessions, but most studies used a much lower threshold for defining ‘receipt’ of therapy which could be attending only one or two sessions. We intentionally excluded data from randomised controlled trials as we wanted to focus on rates of receipt within routine clinical care. However, we included data from a range of other study designs, which added to the heterogeneity of the studies included in the review. This was largely a pragmatic decision, given that studies lie on a spectrum from observational to interventional, rather than these being discrete categories. Service evaluation projects which were further along the spectrum towards the interventional end were unsurprisingly more likely to report higher rates of receipt of therapy. For example, two of the studies reporting the highest rates of CBTp receipt (~ 60%) both reported outcomes from newly set up services for first episode psychosis which included universal access to recommended therapies as part of the care pathway [19, 30], which is not standard across other services.

For future research, there is a need for more data on recommended treatments and implementation of guidelines for schizophrenia and psychosis in middle- and lower-income countries, and from the continents of Asia, South America, and Africa. Mental health care systems differ widely across different countries in terms of how they are funded and delivered [46, 47]. A fully international view must of course take into account cultural, spiritual, and religious differences in how schizophrenia and psychosis are conceptualised in relation to causes, social stigma, and acceptability of psychiatric treatment [48].

## Conclusion

The findings of this review indicate varying rates of receipt of recommended psychological therapies across 9 different countries; however, overall low rates of implementation indicate room for improvement in terms of increasing access to therapies in line with clinical guidelines. The available data were UK-centric, and there were no eligible studies found from the continents of South America, Asia, or Africa, meaning that a truly global picture of provision of psychological therapies for psychosis and schizophrenia is currently lacking.

## Supplementary Information

**Additional file 1. **Database search terms

**Additional file 2: Figure S1.** Pooled prevalence of service-users who were offered CBTp. **Figure S2.** Pooled prevalence of service-users who received CBTp in countries with defined treatment guidelines. **Figure S3.** Pooled prevalence of service-users who received CBTp in countries where treatment guidelines were not available. **Figure S4.** Pooled prevalence of service-users who received CBTp_EI only. **Figure S5.** Pooled prevalence of service-users who received FI_EI only. **Table S1.** Summary of other recommended therapies. **Table S2.** Summary of predictors of therapy receipt.

## Data Availability

The datasets generated and/or analysed during the current study, and STATA analysis script, are available on the Open Science Framework repository, https://doi.org/10.17605/OSF.IO/FSEQM.

## Abbreviations

APA: American Psychiatric Association
CBTp: Cognitive-Behavioural Therapy for Psychosis
DSM-5: Diagnostic and Statistical Manual of Mental Health Disorders 5th edition
DGPPN: German Society of Psychiatry, Psychotherapy and Nervous Diseases
EI: Early Intervention
FI: Family Intervention
ICD-10: International Statistical Classification of Diseases and Related Health Problems
NHS: National Health System
NICE: National Institute for Clinical and Health Excellence
OR: Odds ratio
PORT: Patient Research Outcomes Team
PRISMA: Preferred Reporting Items for Systematic Reviews and Meta-analyses
RANZCP: Royal Australian and New Zealand College of Psychiatry
UK: United Kingdom
USA: United States of America
