# Supplementary material for: International rates of receipt of psychological therapy for psychosis and schizophrenia: systematic review and meta-analysis
Source: Int J Ment Health Syst. 2023 Mar 31;17:8. doi: 10.1186/s13033-023-00576-9 (PMC10064673; doi:10.1186/s13033-023-00576-9)
Supplement: Supplementary file 1 — Additional file 1. Database search terms [file 13033_2023_576_MOESM1_ESM.pdf]

International rates of receipt of psychological therapy for psychosis and schizophrenia: Systematic review and meta-analysis

<u>Additional File 1</u>

1. **<u>Database Search Terms</u>**

International rates of receipt of psychological therapy for psychosis and schizophrenia: Systematic review and meta-analysis

Database Search Terms

PsycInfo

Abstract: schizo* *OR* **Abstract**: psychoses *OR* **Abstract**: psychosis *OR* **Abstract**: psychotic *AND* **Abstract**: "cognitive behavio?r*" OR "psycho* therap*" OR "evidence base*" OR "family intervention" OR "family therapy" OR CBT OR FI OR intervention OR treatment OR guideline* *AND* **Abstract**: implementation *OR* **Abstract**: receipt *OR* **Abstract**: prevalence *OR* **Abstract**: incidence *OR* **Abstract**: referral* *OR* **Abstract**: adherence *OR* **Abstract**: rate* *NOT* **Title**: randomi*ed *AND NOT* **Methodology**: Qualitative Study *NOT* **Methodology**: Clinical Case Study *AND* **Population Group**: Human *AND* **Year**: 2010 *To* 2020

Pubmed

(((schizo*[Title/Abstract] OR psychosis[Title/Abstract] OR psychoses[Title/Abstract] OR psychotic[Title/Abstract]) AND (cognitive-behaviour*[Title/Abstract] OR cognitive-behavior*[Title/Abstract] OR psychological-therap*[Title/Abstract] OR psychotherap*[Title/Abstract] OR psycho-therap*[TItle/Abstract] OR evidence-base*[Title/Abstract] OR family-intervention[Title/Abstract] OR family-therapy[Title/Abstract] OR CBT[Title/Abstract] OR FI[Title/Abstract] OR intervention[Title/Abstract] OR treatment[Title/Abstract] OR guideline*[Title/Abstract])) AND (implementation[Title/Abstract] OR receipt[Title/Abstract] OR prevalence[Title/Abstract] OR incidence[Title/Abstract] OR referral*[Title/Abstract] OR adherence[Title/Abstract] OR rate*[Title/Abstract])) NOT (randomised[Title] OR randomized[Title])

Embase

(**schizo***:ab,ti OR **psychosis**:ab,ti OR **psychoses**:ab,ti) AND (**'cognitive behaviour*'**:ab,ti OR **'cognitive behavior*'**:ab,ti OR **'psychological therap*'**:ab,ti OR **'psychotherap*'**:ab,ti OR **'psycho-therap*'**:ab,ti OR **'evidence base*'**:ab,ti OR **'family intervention'**:ab,ti OR **'family therapy'**:ab,ti OR **cbt**:ab,ti OR **fi**:ab,ti OR **intervention**:ab,ti OR **treatment**:ab,ti OR **guideline***:ab,ti) AND (**implementation**:ab,ti OR **receipt**:ab,ti OR **prevalence**:ab,ti OR **incidence**:ab,ti OR **referral***:ab,ti OR **'patient compliance'**:ab,ti) NOT **randomi*ed**:ti AND [2010-2020]/py AND **'human'**/de NOT **'qualitative research'**/de NOT **'case study'**/de
