# Supplementary material for: International rates of receipt of psychological therapy for psychosis and schizophrenia: systematic review and meta-analysis
Source: Int J Ment Health Syst. 2023 Mar 31;17:8. doi: 10.1186/s13033-023-00576-9 (PMC10064673; doi:10.1186/s13033-023-00576-9)
Supplement: Supplementary file 2 — Additional file 2: Figure S1. Pooled prevalence of service-users who were offered CBTp. Figure S2. Pooled prevalence of service-users who received CBTp in countries with defined treatment guidelines. Figure S3. Pooled prevalence of service-users who received CBTp in countries where treatment guidelines were not available. Figure S4. Pooled prevalence of service-users who received CBTp_EI only. Figure S5. Pooled prevalence of service-users who received FI_EI only. Table S1. Summary of other recommended therapies. Table S2. Summary of predictors of therapy receipt. [file 13033_2023_576_MOESM2_ESM.pdf]

International rates of receipt of psychological therapy for psychosis and schizophrenia: Systematic review and meta-analysis

Additional File 2

1. Figure S1: Pooled prevalence of service-users who were offered CBTp
2. Figure S2: Pooled prevalence of service-users who received CBTp in countries with defined treatment guidelines
3. Figure S3: Pooled prevalence of service-users who received CBTp in countries where treatment guidelines were not available
4. Figure S4: Pooled prevalence of service users who received CBTp EI only
5. Figure S5: Pooled prevalence of service users who received FI EI only
6. Table S1: Summary of other recommended therapies
7. Table S2: Summary of predictors of therapy receipt

Figure S1: Pooled prevalence of service-users who were offered CBTp

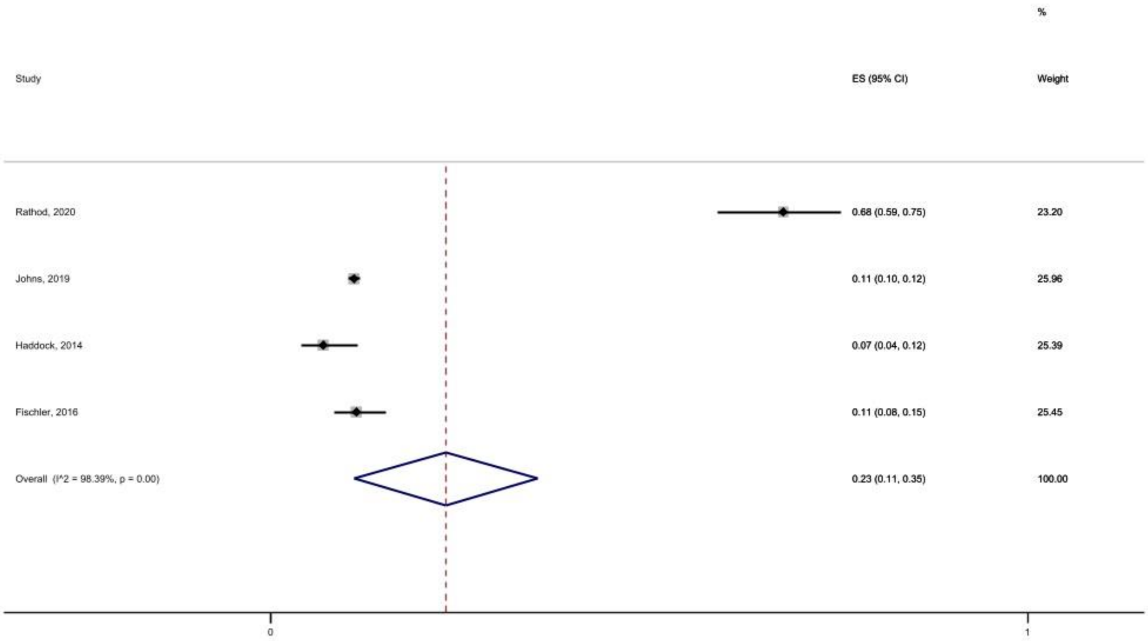

Figure S2: Pooled prevalence of service-users who received CBTp in countries with defined treatment guidelines

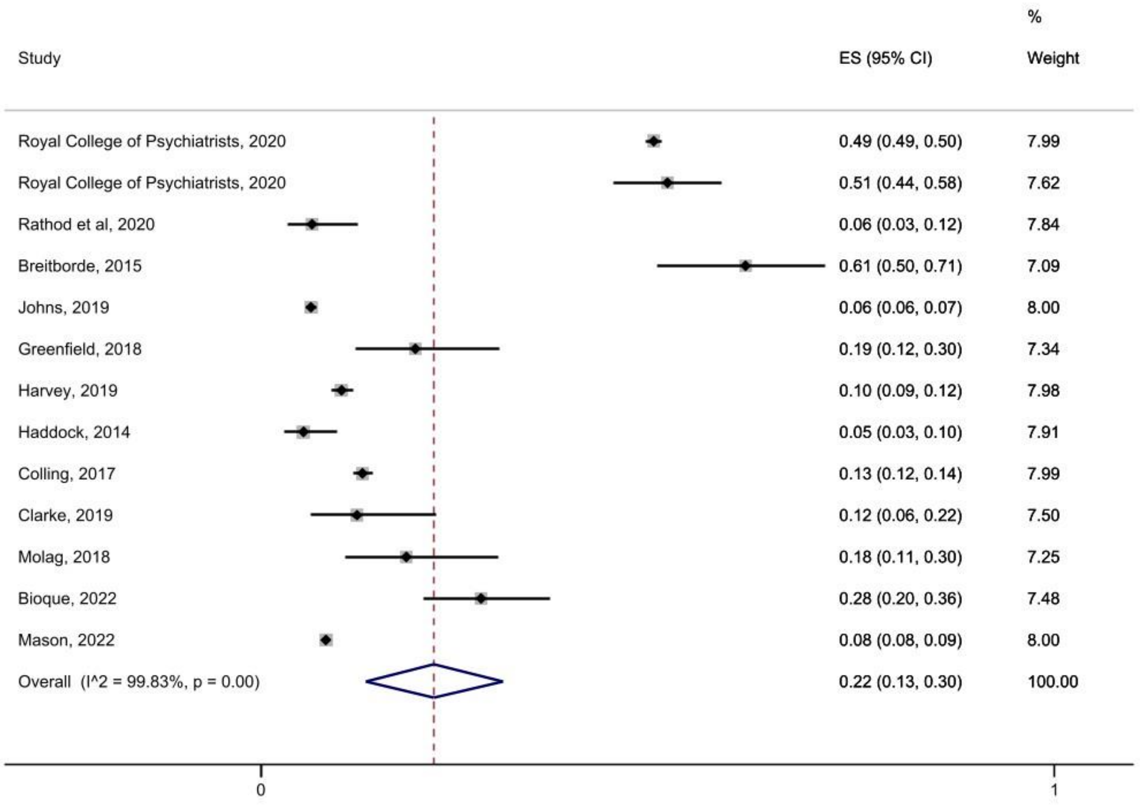

Figure S3: Pooled prevalence of service-users who received CBTp in countries where treatment guidelines were not available

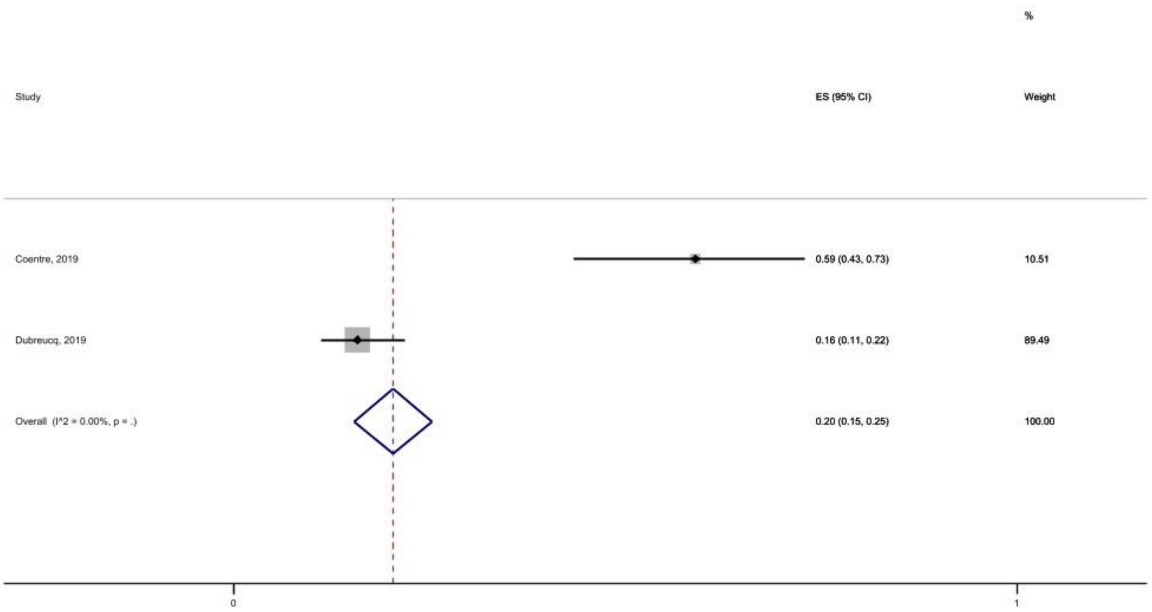

Figure S4: Pooled prevalence of service users who received CBTp EI only

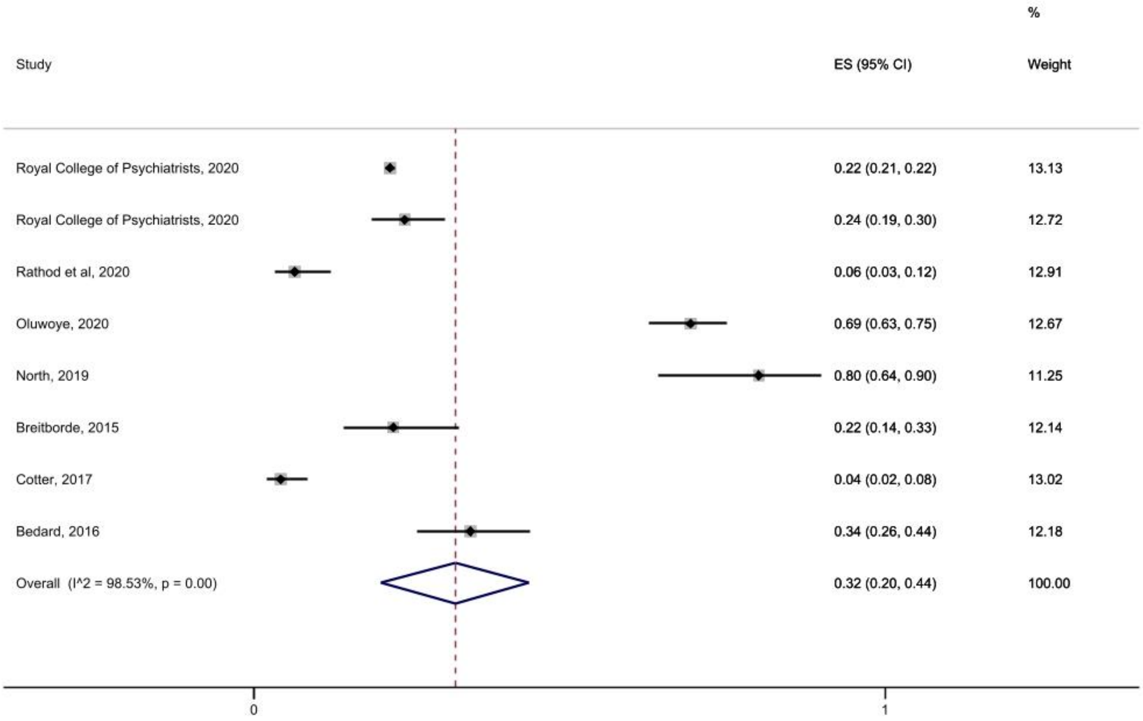

Figure S5: Pooled prevalence of service users who received FI EI only

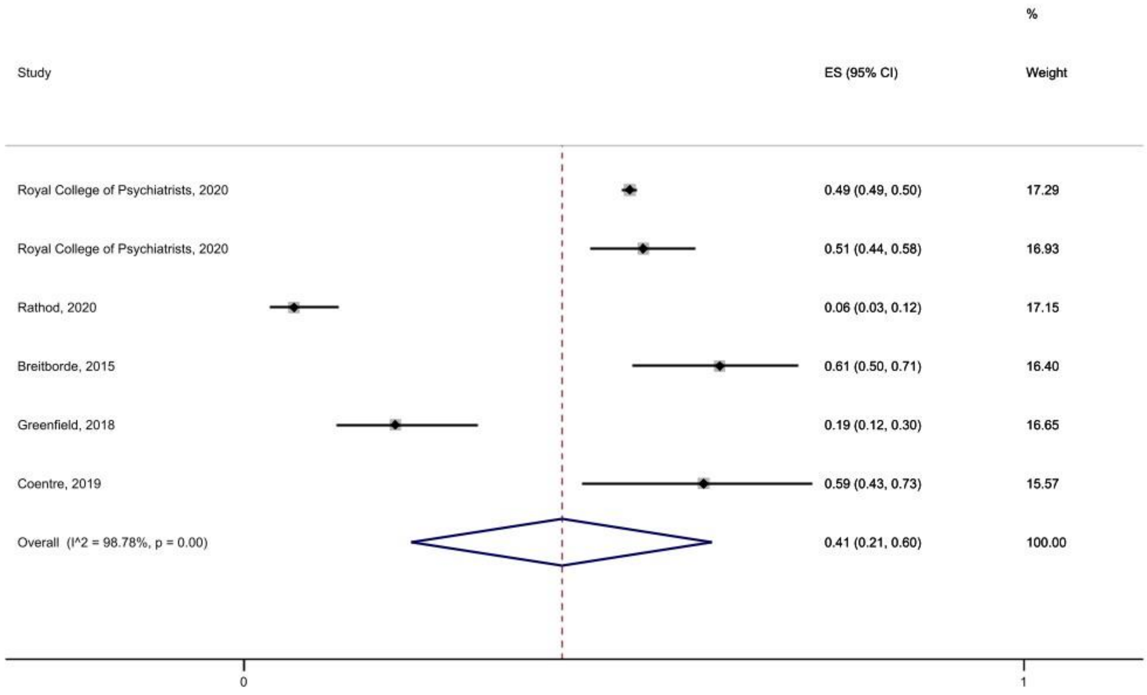

Table S1: Summary of other recommended therapies

STUDY	Other recommended therapy	Numerator	Denominator	Percentage
Addington et al. 2012	Psychotherapy	174	216	81
Bioque et al. 20200	Cognitive Remediation therapy	6	119	5.0
Breitborde et al. 2015	Metacognitive remediation	19	77	24.7
Coleman et al. 2016	Psychotherapy	159	35,812	0.44
Dubreucq et al. 2019	Cognitive remediation therapy	36	183	19.7

Table S2: Summary of predictors of therapy receipt

8.5			Predictor				
**Study**	**Therapy**	**Age**	**Ethnicity**	**Diagnosis**	**Gender**	**Marital Status**	**Service type**
Colling et al. 2017	CBTp	Under 41 - OR = 1.57, 95% CI: 1.01-1.72 compared to Over 41	White - OR = 1.43, 95% CI = 1.10 - 1.85. Other - OR = 1.31, 95% CI = 0.93 - 1.86 compared to Black	Other schizophrenia spectrum - OR = 1.52, 95% CI = 1.05 - 2.20. Schizoaffective disorder - OR = 1.48, 95% CI = 1.11 - 1.98 compared to schizophrenia	Not significant, Female OR = 1.2, 95% CI = 0.94 - 1.54, p = 0.139 compared to Male	Not significant, married/cohabiting - OR = 0.95, 95% CI = 0.63 - 1.44, p=0.809 compared with single/divorced	Early Intervention - OR = 1.98, 95% CI = 1.40 - 2.81 compared to Promoting Recovery
Haddock et al. 2014	CBTp	Not significant, age groups not defined, no data given	Not significant, White/Black/Asian/Mixed/Other, no data given	Other psychosis - OR = 3.75, p = 0.053 compared to schizophrenia	Not significant, gender groups not defined, no data given	OR = 2.29, p = 0.008 Named carer listed when compared with those who did not	
Harvey et al. 2019	CBTp	Not significant, Ages 18-34, 35-65, no data given		Non affective psychosis - OR = 2.51, CI = 1.79-3.52 compared to affective psychosis	Female OR = 2.21, 95% CI = 1.60-3.05 compared to Male	Not significant, In a marital/de facto relationship (no/yes), no data given	
Mason et al. 2022	CBTp	Welch two sample t-test, significant between-group differences (t=15.34, p<0.01) for lower age (M=33.12 SD=11.5) (M=35.88, SD=13.08)		Comorbid depression diagnosis ($\chi2$=87.36), bipolar diagnosis ($\chi2$=71.94), anxiety diagnosis ($\chi2$=118.28)	Not significant, Female compared to Male		
Oluwoye et al. 2020	FI	Not significant, age groups not defined, no data given	Not significant, Latinx/Black/Other, no data given				
